# The role of threat anticipation in the development of psychopathology in adolescence: findings from the SIGMA Study

**DOI:** 10.1007/s00787-022-02048-w

**Published:** 2022-07-29

**Authors:** Isabell Paetzold, Jessica Gugel, Anita Schick, Olivia J. Kirtley, Robin Achterhof, Noemi Hagemann, Karlijn S. F. M. Hermans, Anu P. Hiekkaranta, Aleksandra Lecei, Inez Myin-Germeys, Ulrich Reininghaus

**Affiliations:** 1 Department of Public Mental Health, Central Institute of Mental Health, Medical Faculty Mannheim, Heidelberg University, J5, 68159 Mannheim, Baden-Württemberg Germany; 2 Department of Neuroscience, Center for Contextual Psychiatry, KU Leuven, Leuven, Flanders Belgium; 3 ESRC Centre for Society and Mental Health and Social Epidemiology Research Group, King’s College London, London, UK; 4 Health Service and Population Research Department, Centre for Epidemiology and Public Health, Institute of Psychiatry, King’s College London, Psychology & Neuroscience, London, UK

**Keywords:** Childhood trauma, Bullying, Prodromal psychotic symptoms, Youth mental health

## Abstract

Childhood adversity is associated with psychopathology. First evidence in adults suggests that threat anticipation, i.e., an enhanced anticipation of unpleasant events creating an enduring sense of threat, may be a putative mechanism linking childhood adversity to psychopathology. This study aimed to test the indirect effect of childhood adversity on psychopathology via threat anticipation in a large community sample of adolescents. We measured childhood trauma and bullying victimization (as indicators of childhood adversity), threat anticipation, general psychopathology and prodromal psychotic symptoms in adolescents aged 12–16 years (full sample size *N* = 1682; minimum sample size in the complete case sample *N* = 449) in wave I of the SIGMA study. We found strong evidence that childhood adversity (e.g. childhood trauma, adj. β (aβ) = 0.54, *p* < .001) and threat anticipation (e.g. aβ = 0.36, *p* < .001) were associated with general psychopathology and prodromal psychotic symptoms. Moreover, there was evidence that the association between childhood adversity, general psychopathology and prodromal psychotic symptoms is mediated via pathways through threat anticipation (e.g. childhood trauma, aβ_indirect effect_ = 0.13, *p* < .001). Threat anticipation may be a potential mechanism linking childhood adversity and psychopathology in adolescents.

**Supplementary Information:**

The online version contains supplementary material available at 10.1007/s00787-022-02048-w.

## Introduction

Most mental disorders first emerge during adolescence and young adulthood. Three quarters of all lifetime cases have their onset before the age of 24 [1]. With approximately one-fourth of youth having experienced a mental disorder during the past year [2], mental disorders contribute substantially to the disease burden in young age groups [3]. Trajectories are often characterized by transitional staging processes from relatively mild distress and subclinical symptoms to clinical severity highlighting the potential and relevance of early intervention [1, 4]. In addition, dimensional classification frameworks [5, 6] cutting across traditional boundaries of diagnoses have emerged. However, underlying mechanisms in the development of psychopathology remain unclear, so deepening our understanding of these mechanisms is a crucial step towards improving existing and developing new preventive and early intervention strategies [7].

Converging evidence identified childhood adversity as a risk factor for psychopathology. McLaughlin [8] defines childhood adversity as “experiences that are likely to require significant adaptation by an average child and that represent a deviation from the expectable environment” (p.3), for example, childhood trauma and bullying. Childhood adversity is associated with a heightened risk for mental disorders in youth, but also in later life [9, 10]. Previous research tentatively indicates specificity in the links of different types of childhood adversity with anxiety, affective or personality disorders [11, 12], but not with psychosis [13].

Childhood trauma refers to potentially harmful experiences including sexual, physical and emotional abuse as well as physical and emotional neglect [14]. Previous research indicates a high prevalence in the general population [15], individuals at ultra-high risk for psychosis [16] and with severe mental disorder [13, 17]. Childhood trauma has been shown to be associated with internalizing and externalizing problems [9], psychotic experiences in the general population, the persistence of psychotic symptoms in subclinical and clinical samples, and an increased risk for psychosis [13, 18].

Bullying refers to an individual or a group engaging in hostile behaviour against others who have problems defending themselves[19], for example “teasing, name calling, mockery, threats, harassment, taunting, hazing, social exclusion or rumours”[20] (p.403). With the increasing availability of technologies, cyber bullying (i.e., bullying using technology [21]) has emerged. Experiences of bullying are highly prevalent in youth [22]. Evidence has accumulated linking bullying to general psychopathology [23] and various mental disorders [13, 24]. Moreover, bullying is associated with the later development of psychotic symptoms [25] as well as with higher levels of psychotic experiences in the general population [26].

In summary, childhood adversity has been found to be relevant across a range of psychopathological outcomes [9, 10], tentatively suggesting common, transdiagnostic mechanisms in their development [27]. A putative transdiagnostic mechanism linking childhood adversity and psychopathology may be threat anticipation: Repeated or chronic exposure to adversity may lead to a cognitive bias comprising enhanced anticipation of unpleasant events creating an enduring sense of threat [28–30]. Maladaptive high levels of threat anticipation are postulated as a core factor in the development and maintenance of anxiety disorders [31]. There is evidence for an association between threat anticipation and psychotic experiences in general [32], and especially paranoia [28, 33]. Klippel, et al. [34] showed that the effect of stress on psychotic experiences was mediated via threat anticipation. Further, threat anticipation was associated with more intense psychotic experiences in participants with a first-episode of psychosis and high levels of childhood trauma [35].

The role of threat anticipation has been investigated in several mental disorders, especially psychosis, but to date has not been studied as a putative transdiagnostic mechanism linking childhood adversity and psychopathology. Above-mentioned studies are based on adult samples, so that to date, the role of threat anticipation has not been explored in adolescents yet as a priority target population for prevention and early intervention. Drawing on a large sample of adolescents, the current study aimed to investigate whether of the association between childhood adversity and psychopathology is mediated via pathways through threat anticipation. We tested the following hypotheses (for graphic illustration see Figure S1, online resources):

*H1.* Higher levels of threat anticipation are associated with higher levels of (a) general psychopathology, and (b) prodromal psychotic symptoms (i.e., anomalous experiences and perceived distress).

*H2.* Higher levels of childhood adversity, characterized by childhood trauma and experiences of bullying, are associated with higher levels of (a) general psychopathology, and (b) prodromal psychotic symptoms (i.e., anomalous experiences and perceived distress).

*H3a.* The association between childhood adversity, characterized by childhood trauma and experiences of bullying, and (a) general psychopathology, and (b) prodromal psychotic symptoms (i.e., anomalous experiences and perceived distress) is mediated via pathways through threat anticipation.

In exploratory analyses, we further aimed to examine the specificity of different types of adversity with respect to their association with psychopathology. Furthermore, we sought to investigate the associations between childhood adversity and specific dimensions of psychopathology.

## Methods

### Sample

This study used data from the SIGMA study, a large cohort study with adolescents aged 12 to 16 years conducted in Flanders, Belgium, focusing on the socio-developmental origins of alterations in psychological mechanisms associated with psychopathology [36]. Inclusion criteria were as follows: (1) age of 12 to 16 years, (2) ability to understand the study procedures, (3) adequate command of Dutch language. Written informed consent from at least one caregiver and the adolescent was required. Further details on recruitment, procedures, ethics and consent are described elsewhere [36]. For the current study, cross-sectional data collected as part of wave I was used. Data were collected between January 2018 and May 2019.

## Data collection

### Threat anticipation

Threat anticipation was assessed with a shortened version of the availability test [30, 33]. Participants were asked to predict the likelihood of five threatening events (e.g.’you are being followed by someone’) happening to them in the coming week using a scale of 1 (‘not at all’) to 7 (‘very likely’). In line with previous work, threat anticipation was operationalized as a sum score for the anticipated likelihood of threatening events [33]. Cronbach’s alpha was α = 0.67.

### Childhood adversity

Childhood trauma was assessed using the Juvenile Victimisation Questionnaire (JVQ) [37], a self-report questionnaire comprising 5 subscales (‘any conventional crime’, ‘any child maltreatment’, ‘any peer or sibling victimization’, ‘any sexual victimization’, and ‘any witnessing or indirect victimization’). The full version includes 34 potential victimizations scored dichotomously with 0 (‘no’) and 1 (‘yes’). Twelve-year-old participants completed a shortened version with 25 items (omitting the scale ‘any conventional crime’). Childhood trauma was operationalized as a mean score. The scoring of all composite items was aggregated and divided by the total number of items administered (i.e., 34 items for participants aged 14 and older, 25 items for 12-year old participants). We observed good internal consistency for childhood trauma (*α* = 0.85).

Bullying victimization was assessed with two self-constructed items on severity and frequency devised and included based on an amended questionnaire version of the Retrospective Bullying Interview [36, 38]. Participants were asked to rate their prevalence of bullying on a scale from 0 (‘never’) to 4 (‘often, every week or several times per week’). Bullying severity was reported on a scale from 0 (‘You’ve never been bullied or just a little teased that didn't bother you.’) to 3 (‘Severe, very bad bullying that you have had a lot of trouble with; you have become very upset about this; this has prevented you from daring or wanting to go to some places or people; you have had nightmares about this before.’). Moderate correlations with peer and sibling victimization indicate concurrent validity (*r* = 0.43 for severity and frequency).

### Psychopathology

To assess general psychopathology, we used the Brief Symptom Inventory (BSI-53) [39], a self-report questionnaire consisting of nine subscales measuring different relevant dimensions of mental disorders (somatization, obsessive-compulsive, interpersonal sensitivity, depression, anxiety, hostility, phobic anxiety, paranoid ideation, and psychoticism) and four additional items (appetite, sleeping problems, thoughts about death and dying, and feelings of guilt). Participants are asked to indicate to what extend they experienced the listed symptoms in the last 7 days on a scale ranging from 0 (‘not at all’) to 4 (‘very strong’). The Global Severity Index (GSI), operationalized as the mean score, was used as a measure of general psychopathology. We observed excellent internal consistency for the GSI (*α* = 0.96).

Prodromal psychotic symptoms were assessed using a brief version of the Prodromal Questionnaire (PQ-16), a self-report screening questionnaire for psychosis risk [40]. It comprises 16 items on anomalous experiences rated with ‘yes’/‘no’. If participants confirmed a symptom, perceived distress (‘How much distress did you experience?’) was assessed on a scale ranging from 0 (‘none’) to 3 (‘severe’). If participants negate a symptom, the perceived distress variable was not presented. The PQ-16 was omitted for 12-year-old participants. We used a total score (i.e., number of items answered with ‘yes’) as a measure of anomalous experiences and a sum score of the perceived distress by any given prodromal psychotic symptoms. For anomalous experiences, we observed satisfying internal consistency (*α* = 0.76).

### Sociodemographic and intellectual characteristics

Age, gender and self-reported ethnicity were assessed as sociodemographic characteristics. In the assessment of self-reported ethnicity, participants were asked to indicate to which groups other than Belgian they felt related to, multiple answers were allowed. In addition, the THINC-it application [41] was used to screen for impairments in cognitive functioning.

## Statistical analysis

The study was registered on the open science framework prior to accessing the datahttps://osf.io/92e3w (https://osf.io/92e3w), deviations from the preregistration are made transparent in online resource 2. As the data have a multilevel structure with multiple students (level-1) nested within schools (level-2), the ‘mixed’ command in Stata 16 [42] was used to fit two-level, linear mixed models. First, we included threat anticipation as an independent variable and general psychopathology (H1a) and prodromal psychotic symptoms (H1b) as outcome variables. Second, we included childhood trauma, bullying prevalence and severity as independent variables and general psychopathology (H2a) and prodromal psychotic symptoms (H2b) as outcome variables. Third, we performed mediation analysis using the ‘gsem’ command to investigate the indirect effects of childhood trauma, bullying prevalence and severity on general psychopathology (H3a) and prodromal psychotic symptoms (H3b) via pathways through threat anticipation. The total effect of childhood adversity on outcomes was apportioned into a direct effect and an indirect effect through threat anticipation. The indirect effect was computed using the product of coefficients strategy. We computed the proportion mediated (i.e., the ratio of the indirect effect to the total effect) as a measure of effect size.

In exploratory analyses (see Tables S1-S4, online resources), we used specific types of trauma and bullying as independent variables and general psychopathology and prodromal psychotic symptoms as outcome variables. In addition, we examined whether childhood trauma and bullying were associated with different dimensions of psychopathology (operationalized by the BSI subscales) and investigated differential indirect effects for dimensions of prodromal psychotic symptoms.

We used random intercept and slope models and applied restricted maximum likelihood (H1 and H2) or maximum likelihood estimation (H3), allowing for the use of all available data under the relatively unrestrictive assumption that data are missing at random and all variables associated with missing values are included in the model (see Table S5, online resources for an overview of missing values) [43, 44]. Results were bootstrapped with 1.000 repetitions. For sensitivity analyses with restrictions on missing values, exclusion of outliers and robust standard errors see Tables S6-S17, online resources. All analyses were adjusted for potential confounders (i.e., age, gender, self-reported ethnicity, and deviations in cognitive functioning; for unadjusted analyses see Tables S18–S20, online resources). We corrected for multiple testing within each hypothesis to reduce the probability of type I errors as a consequence of the number of tests performed. We used Simes’ correction, a modified version of the more conservative Bonferroni correction in case of dependent hypotheses given significance tests in the current analyses were not independent [45]. With the Simes’ correction, the most significant p-value is tested against *α* = 0.05/n (total number of tests), the second most significant p-value is tested against *α* = 0.05/(n − 1), etc. Simes-corrected significant results are highlighted with an asterisk (*) in text and tables. The study is reported based on STROBE criteria for reporting for cross-sectional studies [46].

## Results

### Basic sample characteristics

Table 1 displays relevant sample characteristics. The sample comprised *N* = 1,82 adolescents with a mean age of 13.4 years (*SD* = 1.47); 63% were girls. The majority (70%) reported to be Belgian only, 9% reported Moroccan and 5% Turkish ethnicity in addition to Belgian. The lifetime prevalence of trauma ranged from 21% (sexual victimization) to 65% (peer or sibling victimization). In addition, 58% of all participants reported to have experienced bullying victimization at least once. A majority of 71% reported at least one prodromal psychotic symptom and 28% of all participants reached the cutoff for relevant prodromal psychotic symptomatology (i.e., six symptoms). The mean level of general psychopathology was *M* = 0.82 (*SD* = 0.59).

**Table 1:** Basic sample characteristics of the full sample

	Full sample
	*(N* = *1682)*^*a*^
Age (years), mean (*SD*)	13.4 (1.47)
Gender, *N* (%)	
Male	619 (37%)
Female	1.053 (63%)
Other	6 (0.4%)
Self-reported ethnicity, *N* (%)^b^	
Only Belgian	1.183 (70%)
Moroccan	146 (9%)
Turkish	84 (5%)
Berbers	65 (4%)
Italian	42 (3%)
Polish	21 (1%)
Kurdish	16 (1%)
Other	238 (14%)
Lifetime prevalence of at least one experience of childhood adversity, *N* (%)	
Conventional crime^c^	512 (30%)
Indirect victimization	783 (47%)
Child maltreatment	596 (35%)
Peer or sibling victimization	1,100 (65%)
Sexual victimization	348 (21%)
Bullying prevalence	707 (58%)
Cyber bullying prevalence	367 (30%)
Physical bullying prevalence	613 (50%)
Threat anticipation, mean (*SD*)	10.24 (6.10)
Lifetime prevalence of prodromal symptoms, *N* (%)^c^	
At least one symptom	464 (71%)
At least six symptoms (cutoff)^d^	181 (28%)
General psychopathology, mean (*SD*)	0.82 (0.59)

^a^Full sample size was 1682, sample sizes varied over the different scales due to missing values

^b^Identification as Belgian was assumed, children were asked to state all other nationalities they identify with, multiple answers were allowed on this scale, so that the number does not add up to the full sample of 1682 participants

^c^This scale was not answered by participants in the first year (~ 12 years old)

^d^A cutoff score of ≥ 6 on the PQ-16 identifies people at ultra-high-risk for developing psychosis with a sensitivity and specificity of 87% each [40]

### The association between threat anticipation and psychopathology (H1)

As displayed in Table 2, threat anticipation predicted psychopathology, such that higher levels of threat anticipation were associated with more severe general psychopathology (adj. β (aβ) = 0.36, 95% CI = 0.28 – 0.41, *p* < 0.001*, *n* = 3 for Simes’ correction), more anomalous experiences (aβ = 0.28, 95% CI = 0.17 – 0.38, *p* < 0.001*, n = 3 for Simes’ correction) and higher levels of perceived distress (aβ = 0.36, 95% CI = 0.24 – 0.47, *p* < 0.001*, *n* = 3 for Simes’ correction).

**Table 2:** General psychopathology and prodromal symptoms predicted by threat anticipation^a^

	General psychopathology			Prodromal symptoms					
				Anomalous experiences			Perceived distress		
	aβ (CI)	*p*	*N*	aβ (CI)	*p*	*N*	aβ (CI)	*p*	*N*
Threat anticipation	0.36 (0.28 – 0.41)	< .001*	1.384	0.28 (0.17 – 0.38)	< .001*	607	0.36 (0.24 – 0.47)	< .001*	607

aβ = results adjusted for age, gender, self-reported ethnicity, and cognitive deviance. * = statistically significant after Simes’correction with *n* = 3

^a^Results were bootstrapped with 1.000 repetitions

### The association between childhood adversity and psychopathology (H2)

Table 3 shows the association between childhood adversity and psychopathology. Individuals reporting higher levels of childhood trauma (aβ = 0.54, 95% CI = 0.48 – 0.61, *p* < 0.001*, *n* = 9 for Simes’ correction) as well as higher prevalence (aβ = 0.36, 95% CI = 0.28 – 0.42, *p* < 0.001*, *n* = 9 for Simes’ correction) and severity of bullying (aβ = 0.42, 95% CI = 0.35 – 0.48, *p* < 0.001*, *n* = 9 for Simes’ correction) experienced more severe general psychopathology. Moreover, participants with higher levels of childhood trauma reported more anomalous experiences (aβ = 0.32, 95% CI = 0.24 – 0.41, *p* < 0.001*, *n* = 9 for Simes’ correction) and higher levels of perceived distress (aβ = 0.34, 95% CI = 0.25 – 0.42, *p* < 0.001*, *n* = 9 for Simes’ correction). Higher bullying prevalence and severity were associated with more anomalous experiences (e.g., prevalence: aβ = 0.23, 95% CI = 0.13 – 0.33, *p* < 0.001*, *n* = 9 for Simes’ correction) and higher levels of perceived distress (e.g., severity: aβ = 0.28, 95% CI = 0.14 – 0.37, *p* < 0.001*, *n* = 9 for Simes’ correction).

**Table 3:** General psychopathology and prodromal symptoms predicted by childhood adversity^a^

	General psychopathology			Prodromal symptoms					
				Anomalous experiences			Perceived distress		
	aβ (CI)	*p*	*N*	aβ (CI)	*p*	*N*	aβ (CI)	*p*	*N*
Childhood trauma	0.54 (0.48 – 0.61)	< .001*	1,239	0.32 (0.24 – 0.41)	< .001*	563	0.34 (0.25 – 0.42)	< .001*	563
Bullying prevalence	0.36 (0.28 – 0.42)	< .001*	1,045	0.23 (0.13 – 0.33)	< .001*	449	0.24 (0.14 – 0.34)	< .001*	449
Bullying severity	0.42 (0.35 – 0.48)	< .001*	1,059	0.26 (0.15 – 0.35)	< .001*	452	0.28 (0.14 – 0.37)	< .001*	452

aβ = results adjusted for age, gender, cultural identification, and cognitive deviance * = statistically significant after Simes’ correction with *n *= 9

^a^Results were bootstrapped with 1.000 repetitions

### The association between childhood adversity and psychopathology is mediated via pathways through threat anticipation (H3)

Table 4 shows findings on total, direct and indirect effects of childhood adversity and threat anticipation on psychopathology. For the associations between childhood adversity and general psychopathology we observed evidence for mediation effects via threat anticipation (e.g., childhood trauma, indirect effect: aβ = 0.13, 95% CI = 0.09 – 0.16, *p* < 0.001*, *n* = 9 for Simes’ correction). In addition, there was evidence for mediation effects via threat anticipation for the associations between childhood adversity and prodromal psychotic symptoms (e.g. childhood trauma, indirect effect on perceived distress: aβ = 0.08, 95% CI = 0.03 – 0.13, *p* < 0.001*, *n* = 9 for Simes’ correction). The pathway via threat anticipation (i.e., the proportion mediated) accounted for 12–25% of the total effect.

**Table 4:** The association between childhood adversity and psychopathology is mediated via pathways through threat anticipation^a^

	General psychopathology				Prodromal symptoms							
					Anomalous experiences				Perceived distress			
	aβ (CI)	*p*	*N*_*min*_	*P*_*M*_	aβ (CI)	*p*	*N*_*min*_	*P*_*M*_	aβ (CI)	*p*	*N*_*min*_	*P*_*M*_
Childhood trauma			1.239				563				563	
Total effect	0.55 (0.49 – 0.61)	< 0.001			0.31 (0.23 – 0.42)	< 0.001			0.33 (0.25 – 0.43)	< 0.001		
Direct effect	0.42 (0.35 – 0.49)	< 0.001			0.26 (0.17 – 0.36)	< 0.001			0.25 (0.16 – 0.36)	< 0.001		
Indirect effect	0.13 (0.09 – 0.16)	< 0.001*		0.24	0.05 (0.01 – 0.09)	0.008*		0.16	0.08 (0.03 – 0.13)	< 0.001*		0.24
Bullying prevalence			1.045				449				449	
Total effect	0.35 (0.29 – 0.41)	< 0.001			0.24 (0.15 – 0.32)	<0.001			0.20 (0.09 – 0.30)	< 0.001		
Direct effect	0.30 (0.24 – 0.37)	< 0.001			0.20 (0.12 – 0.29)	< 0.001			0.20 (0.09 – 0.30)	< 0.001		
Indirect effect	0.05 (0.03 – 0.07)	< 0.001*		0.14	0.04 (0.02 – 0.07)	0.001*		0.17	0.05 (0.03 – 0.09)	< 0.001*		0.25
Bullying severity			1.059				452				452	
Total effect	0.41 (0.35 – 0.47)	< 0.001			0.27 (0.18 – 0.38)	<0.001			0.29 (0.20 – 0.40)	< 0.001		
Direct effect	0.36 (0.31 – 0.43)	< 0.001			0.24 (0.14 – 0.34)	< .001			0.25 (0.15 – 0.36)	< 0.001		
Indirect effect	0.05 (0.03 – 0.07)	< 0.001*		0.12	0.04 (0.02 – 0.07)	0.001*		0.15	0.05 (0.02 – 0.08)	< 0.001*		0.17

aβ = results adjusted for age, gender, self-reported ethnicity, and cognitive deviance. *N*_*min*_ = Due to varying numbers of missing values, different paths of the mediation analyses comprised varying sample sizes. Therefore, the minimum sample size is displayed here. *P*_*M*_ = proportion mediated

^a^Results were bootstrapped with 1.000 repetitions

* = Statistically significant after Simes’ correction with *n* = 9

### Exploratory analyses

Results of exploratory analyses are displayed in Tables S1–S4, online resources. Examining specific types of childhood trauma, we observed variation in the magnitude of associations with general psychopathology (indicated by confidence intervals not including point estimates). We found a larger association with peer or sibling victimization (aβ = 0.49, 95% CI = 0.39 – 0.59, *p* < 0.001) in comparison to sexual or indirect victimization (e.g., aβ = 0.26, 95% CI = 0.19 – 0.34, *p* < 0.001).

Examining specific dimensions of psychopathology, we found an especially strong association between childhood trauma and paranoid ideation (aβ = 0.55, 95% CI = 0.49 – 0.61, *p* < 0.001). Examining differential mediation effects, we found evidence for a mediation effect threat anticipation for the association between childhood trauma and delusions (indirect effect: aβ = 0.06, 95% CI = 0.02 – 0.09, *p* = 0.004), but not for the association between childhood trauma and hallucinations (indirect effect: aβ = 0.04, 95% CI = 0.00 – 0.07, *p* = 0.063). However, there was evidence for mediation effects of threat anticipation for the association between bullying prevalence and severity and hallucinations and delusions.

## Discussion

### Main findings

We found evidence that threat anticipation was associated with psychopathology (H1). In addition, experiences of childhood adversity were associated with psychopathology (H2). We observed medium to large associations with childhood trauma and small to medium associations with bullying. Moreover, there was evidence for mediation effects via pathways through threat anticipation for the associations between childhood adversity and psychopathology (H3). In exploratory analyses, we found some evidence for differential associations of specific types of childhood adversity and specific dimensions of psychopathology.

### Methodological considerations

The reported findings should be interpreted in the light of several methodological considerations as follows: First, the cross-sectional design should be taken into account. As temporal precedence is an important criterion of causality [47], the current study focuses on reporting associations and longitudinal designs are needed to further strengthen evidence on the role of threat anticipation. However, as SIGMA is a cohort study, it may be possible to further explore temporal associations with data from future waves of data collection. Second, limitations regarding the data structure should be considered. Measures of psychopathology and childhood adversity were affected by missing values. However, missing values on the perceived distress score are inherent to the instrument used. In addition, sensitivity analyses in restricted samples (see Tables S6–S14, online resources) indicated a similar pattern of findings. Caregivers’ reports were affected by a low response rate and it was not possible to control for social disadvantage. Therefore, we adjusted the analyses for adolescents’ self-reports of known a priori confounders (i.e., age, gender, ethnicity, and deviances in cognitive functioning). In addition, unmeasured confounders (e.g. polygenic risk) may have influenced the reported findings. Third, statistical limitations should be evaluated critically. The number of analyses performed may have resulted in multiple testing problems. In order to control for type I error, results were corrected using the Simes’ method [45] within each hypothesis. Data were left-skewed on several scales, as one would expect in a community sample. In addition, scatter plots revealed slightly pronounced heteroscedasticity for some tests. Sensitivity analyses based on univariate outlier analyses for skewed data [48] and robust standard errors replicated the pattern of findings (see Tables S15-S17, online resources).

### Comparison to previous research

Consistent with previous research we found evidence for an association between childhood adversity and psychopathology in a large community sample of adolescents [9, 49]. The current study extends findings on threat anticipation [29, 32] by showing an association with general psychopathology and prodromal psychotic symptoms. Examining momentary processes, Klippel et al. [34] found that the effect of stress on psychotic experiences was mediated by threat anticipation. The current study broadens the perspective by elucidating the role of threat anticipation as a potential mediator in a larger context linking childhood adversity and psychopathology. In line with previous suggestions of transdiagnostic risk and resilience mechanisms [27, 35] and a recent review on cognitive mediators [50], the partial mediation indicates that threat anticipation may be one of multiple mechanisms underlying this association. Future research should, therefore, examine threat anticipation in combination with other putative mechanisms.

Exploratory analyses demonstrated an especially strong association of childhood trauma and paranoid ideation. In line with the model of psychosis [51], the current findings underscore the relevance of childhood trauma as a potential risk factor in the development of psychotic experiences. In addition, exploratory analyses indicated a potential mediating effect of threat anticipation for the association between childhood trauma and delusions, but not for the association between childhood trauma and hallucinations. These findings are consistent with the hypothesis of different pathways from childhood trauma to hallucinations and from childhood trauma to delusions [28]: For hallucinations, childhood trauma is expected to cause unwanted, intrusive cognitions which, in interaction with dysfunctional, metacognitive beliefs and poor source monitoring, then cause hallucinations. The hypothesized pathway from childhood trauma to delusions or paranoid beliefs is postulated to operate via externalizing explanatory bias and threat anticipation.

## Conclusion

Taken together, our findings underscore the relevance of threat anticipation as putative transdiagnostic mechanism linking childhood adversity with psychopathology in adolescents. Threat anticipation may, therefore, be a potential transdiagnostic target mechanism in the development of prevention and early intervention [7]. Future research could elaborate on this by applying a longitudinal approach. A first example of an intervention targeting threat anticipation as a candidate mechanism is EMIcompass, a hybrid compassion-focused intervention to enhance resilience in help-seeking youth, which is currently tested in an exploratory randomized controlled trial [52].

### Supplementary Information

Below is the link to the electronic supplementary material.

Supplementary file1 (DOCX 193 KB)
