# Supplementary material for: The role of threat anticipation in the development of psychopathology in adolescence: findings from the SIGMA Study
Source: Eur Child Adolesc Psychiatry. 2022 Jul 29;32(11):2119–27. doi: 10.1007/s00787-022-02048-w (PMC10576675; doi:10.1007/s00787-022-02048-w)
Supplement: Supplementary file 1 — Supplementary file1 (DOCX 193 KB) [file 787_2022_2048_MOESM1_ESM.docx]

**Online resources**

*Article Title:* The role of threat anticipation in the development of psychopathology in adolescence: Findings from the SIGMA Study

*Journal:* European Child & Adolescent Psychiatry

*Authors:* Isabell Paetzold^1^, Jessica Gugel^1^, Anita Schick^1^, Olivia J. Kirtley^2^, Robin Achterhof^2^, Noemi Hagemann^2^, Karlijn S. F. M. Hermans^2^, Anu P. Hiekkaranta^2^, Aleksandra Lecei^2^, Inez Myin-Germeys^2^, Ulrich Reininghaus^1, 3, 4^

1 Department of Public Mental Health, Central Institute of Mental Health, Medical Faculty Mannheim, Heidelberg University, Mannheim, Baden-Württemberg, Germany

2 Department of Neuroscience, Center for Contextual Psychiatry, KU Leuven, Leuven, Flanders, Belgium

3 ESRC Centre for Society and Mental Health and Social Epidemiology Research Group, King's College London, London, London, UK

4 Health Service and Population Research Department, Centre for Epidemiology and Public Health, Institute of Psychiatry, Psychology & Neuroscience, King's College London, London, London, UK

**Correspondence**

Isabell Paetzold

Department of Public Mental Health, Central Institute of Mental Health, Central Institute of Mental Health, Medical Faculty Mannheim, Heidelberg University, Mannheim

J5, 68159 Mannheim, Germany;

Tel: +49 621 1703-2370; Fax: 0621 1703-801930; E-mail: isabell.paetzold@zi-mannheim.de

[**1.** **Graphic illustration of the hypotheses** 3](#_Toc82164276)

[**2.** **Transparent changes document** 4](#_Toc82164277)

[**3.** **Exploratory analyses** 5](#_Toc82164278)

[**3.1.** **The association of the specific types of childhood trauma with general psychopathology and prodromal symptoms** 5](#_Toc82164279)

[**3.2.** **The association of specific types of bullying with general psychopathology and prodromal symptoms** 6](#_Toc82164280)

[**3.3.** **The association of childhood adversity with different dimensions of psychopathology** 7](#_Toc82164281)

[**3.4.** **Threat anticipation as a mediator of the association of childhood adversity with dimensions of prodromal symptoms** 8](#_Toc82164282)

[**4.** **Overview of missing values** 9](#_Toc82164283)

[**5.** **Sensitivity analyses** 10](#_Toc82164284)

[**5.1.** **Sensitivity analyses with restrictions on missing values** 10](#_Toc82164285)

[5.1.1. Hypothesis 1: The association of threat anticipation and psychopathology – with restrictions on missing values 10](#_Toc82164286)

[5.1.2. Hypothesis 2: The association of childhood adversity and psychopathology – with restrictions on missing values 10](#_Toc82164287)

[5.1.3. Hypothesis 3: Threat anticipation as a mediator of the association of childhood adversity and psychopathology – with restrictions on missing values 11](#_Toc82164288)

[**5.2.** **Sensitivity analyses after exclusion of outliers** 12](#_Toc82164289)

[5.2.1. Hypothesis 1: The association of threat anticipation and psychopathology – after exclusion of outliers 12](#_Toc82164290)

[5.2.2. Hypothesis 2: The association of childhood adversity and psychopathology – after exclusion of outliers 13](#_Toc82164291)

[5.2.3. Hypothesis 3: Threat anticipation as a mediator of the association between childhood adversity and psychopathology – after exclusion of outliers 14](#_Toc82164292)

[**5.3.** **Sensitivity analyses with restriction on missing values and after exclusion of outliers** 15](#_Toc82164293)

[5.3.1. Hypothesis 1: The association of threat anticipation and psychopathology – with restrictions on missing values and after exclusion of outliers 15](#_Toc82164294)

[5.3.2. Hypothesis 2: The association of childhood adversity and psychopathology – with restrictions on missing values and after exclusion of outliers 16](#_Toc82164295)

[5.3.3. Hypothesis 3: Threat anticipation as a mediator of the association between childhood adversity and psychopathology – with restrictions on missing values and after exclusion of outliers 17](#_Toc82164296)

[**5.4.** **Sensitivity analyses with robust standard errors** 18](#_Toc82164297)

[5.4.1. Hypothesis 1: The association of childhood adversity and psychopathology – with robust standard errors 18](#_Toc82164298)

[5.4.2. Hypothesis 2: The association of childhood adversity and psychopathology – with robust standard errors 19](#_Toc82164299)

[5.4.3. Hypothesis 3: Threat anticipation as a mediator of the association between childhood adversity and psychopathology – with robust standard errors 20](#_Toc82164300)

[**6.** **Unadjusted analyses** 21](#_Toc82164301)

[**6.1.** **Hypothesis 1: The association of threat anticipation and psychopathology – unadjusted** 21](#_Toc82164302)

[**6.2.** **Hypothesis 2: The association of childhood adversity and psychopathology – unadjusted** 21](#_Toc82164303)

[**6.3.** **Hypothesis 3: Threat anticipation as a mediator of the association between childhood adversity and psychopathology** – **unadjusted** 22](#_Toc82164304)

[**7.** **References** 23](#_Toc82164305)

# **Graphic illustration of the hypotheses**

**Figure S1**

*Graphic illustration of the hypotheses tested*


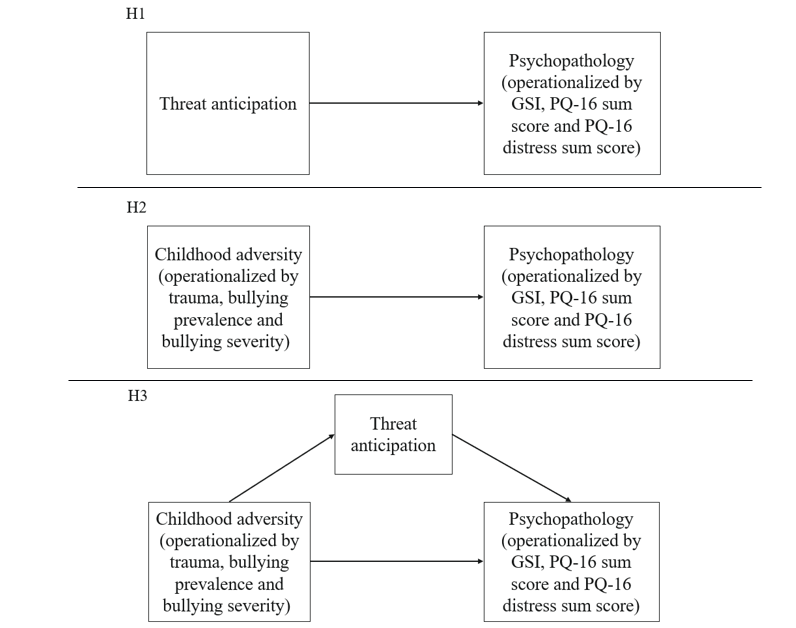


# **Transparent changes document**

This study was registered at the OSF website before accessing the data (<https://osf.io/92e3w>). We made to following deviations:

1. We performed multilevel analyses taking into account schools as a variable of higher level.
2. We calculated a proportion mediated
3. Caregivers’ reports were strongly affected by missing values. Therefore, we were not able to compute an index of social disadvantage. Instead, we adjusted for known a priori confounders (age, gender, self-reported ethnicity, and deviations in cognitive functioning).
4. To adjust for multiple testing, we used Simes correction (Simes, 1986).
5. We performed additional exploratory analyses exploring differential effects on hallucinations and delusions.

# **Exploratory analyses**

## **The association of the specific types of childhood trauma with general psychopathology and prodromal symptoms**

**Table S1**

|  | General psychopathology | | | Prodromal symptoms | | | | | |
| --- | --- | --- | --- | --- | --- | --- | --- | --- | --- |
|  |  |  |  | Anomalous experiences | | | Perceived distress | | |
|  | aβ (CI) | *p* | *N* | aβ (CI) | *p* | *N* | aβ (CI) | *p* | *N* |
| **Childhood trauma** | | | | | | | | | |
| Conventional crime | 0.39  (0.30 – 0.48) | < .001 | 530 | 0.28  (0.16 – 0.39) | < .001 | 530 | 0.29 (0.18 – 0.39) | < .001 | 530 |
| Indirect victimization | 0.26 (0.19 – 0.34) | < .001 | 530 | 0.22 (0.14 – 0.30) | < .001 | 530 | 0.20 (0.11 – 0.30) | < .001 | 530 |
| Child maltreatment | 0.31  (0.23 – 0.38)^a^ | < .001 | 530 | 0.23  (0.14 – 0.33) | < .001 | 530 | 0.25  (0.14 – 0.35) | < .001 | 530 |
| Peer or sibling victimization | 0.49  (0.39 – 0.59) | < .001 | 530 | 0.30  (0.18 – 0.43) | < .001 | 530 | 0.30  (0.19 – 0.42) | < .001 | 530 |
| Sexual victimization | 0.27 (0.20 – 0.34) | < .001 | 530 | 0.21 (0.11 – 0.31) | < .001 | 530 | 0.26 (0.14 – 0.38) | < .001 | 530 |

*Exploratory analyses of the association of the different trauma subscales with general psychopathology and prodromal symptoms*

*Note*. Childhood trauma assessed with the JVQ General psychopathology assessed with the BSI-53. Prodromal symptoms assessed with the PQ-16. To ensure comparability, a constant sample size was used for all analyses displayed. aβ = results adjusted for age, gender, self-reported ethnicity, and cognitive deviance. CI =95% confidence interval. ^a^ = the random intercept and random slope model cloud not be estimated, so the values are estimated with a random intercept model for this test.

## **The association of specific types of bullying with general psychopathology and prodromal symptoms**

**Table S2**

|  | General psychopathology | | | Prodromal symptoms | | | | | |
| --- | --- | --- | --- | --- | --- | --- | --- | --- | --- |
|  |  |  |  | Anomalous experiences | | | Perceived distress | | |
|  | aβ (CI) | *p* | *N* | aβ (CI) | *p* | *N* | aβ (CI) | *p* | *N* |
| Physical bullying | 0.38 (0.26 – 0.49) | < .001 | 408 | 0.28  (0.13 – 0.42) | < .001 | 408 | 0.29  (0.15 – 0.43) | < .001 | 408 |
| Cyber bullying | 0.33  (0.25 – 0.42) | < .001 | 408 | 0.20  (0.10 – 0.30) | < .001 | 408 | 0.22  (0.13 – 0.31) | < .001 | 408 |

*Exploratory analyses of the association of physical vs. cyber bullying with general psychopathology and prodromal symptoms*

*Note*. General psychopathology assessed with the BSI-53. Prodromal symptoms assessed with the PQ-16. To ensure comparability, a constant sample size was used for all analyses displayed. aβ = results adjusted for age, gender, self-reported ethnicity, and cognitive deviance. CI = 95% confidence interval.

and cognitive deviance. CI = 95% confidence interval.

## **The association of childhood adversity with different dimensions of psychopathology**

**Table S3**

|  | | Somatization | | Obsession-compulsion | Interpersonal sensitivity | | | Depression | Anxiety | | Hostility | | | Phobic anxiety | Paranoid ideation | | Psychoticism | |  |
| --- | --- | --- | --- | --- | --- | --- | --- | --- | --- | --- | --- | --- | --- | --- | --- | --- | --- | --- | --- |
|  | aβ (CI) | | aβ (CI) | | | aβ (CI) | aβ (CI) | | | aβ (CI) | | aβ (CI) | aβ (CI) | | | aβ (CI) | | aβ (CI) | |
| Childhood trauma | 0.40  (0.33 – 0.46)^a^ | | 0.42  (0.35 – 0.49) | | | 0.37 (0.29 – 0.44) | 0.44 (0.35 – 0.53) | | | 0.43 (0.37 – 0.50) | | 0.47 (0.41 – 0.53) | 0.32 (0.24 – 0.39) | | | 0.55 (0.49 – 0.61) | | 0.44 (0.36 – 0.51) | |
| Bullying prevalence | 0.27 (0.20 – 0.34) | | 0.29 (0.21 – 0.36) | | | 0.31 (0.24 – 0.38) | 0.36 (0.30 – 0.42)^a^ | | | 0.28 (0.21 – 0.35) | | 0.24 (0.17 – 0.30) | 0.20 (0.13 – 0.27) | | | 0.32 (0.25 – 0.39) | | 0.30 (0.21 – 0.39) | |
| Bullying severity | 0.33 (0.24 – 0.41) | | 0.33 (0.25 – 0.42) | | | 0.37 (0.29 – 0.44) | 0.42 (0.33 – 0.51) | | | 0.36 (0.28 – 0.43) | | 0.28 (0.21 – 0.34) | 0.27 (0.19 – 0.35) | | | 0.37 (0.30 – 0.43) | | 0.36 (0.30 – 0.43)^a^ | |

*Exploratory analyses of the association of childhood adversity (i.e., childhood trauma, bullying prevalence and bullying severity) with the different dimensions of psychopathology*

*Note*. Childhood trauma assessed with the JVQ. Dimensions of psychopathology assessed with the BSI-53. To ensure comparability, a constant sample size was used for all analyses displayed, *N* for all tests = 928. aβ = results adjusted for age, gender, self-reported ethnicity, and cognitive deviance. CI = 95% confidence interval. All *p*-values < .001. ^a^ = the random intercept and random slope model cloud not be estimated, so the values are estimated with a random intercept model for this test.

## **The indirect effect of childhood adversity on dimensions of prodromal symptoms via pathways through threat anticipation**

**Table S4**

|  | Prodromal symptoms | | | | | | | |
| --- | --- | --- | --- | --- | --- | --- | --- | --- |
|  | Hallucinations | | | | Delusions | | | |
|  | aβ (CI) | *p* | *N_min_* | *P_M_* | aβ (CI) | *p* | *N_min_* | *P_M_* |
| **Childhood trauma** |  |  | 563 |  |  |  | 563 |  |
| Total effect | 0.31 (0.24 – 0.38) | < .001 |  |  | 0.26 (0.19 – 0.33) | < .001 |  |  |
| Direct effect | 0.27 (0.20 – 0.35) | < .001 |  |  | 0.20 (0.12 – 0.28) | < .001 |  |  |
| Indirect effect | 0.04 (0.00 – 0.07) | .063 |  | 0.13 | 0.06 (0.02 – 0.09) | .004 |  | 0.23 |
| **Bullying prevalence** |  |  | 449 |  |  |  | 449 |  |
| Total effect | 0.23 (0.15 – 0.32) | < .001 |  |  | 0.22 (0.13 – 0.31) | < .001 |  |  |
| Direct effect | 0.20 (0.11 – 0.28) | < .001 |  |  | 0.19 (0.10 – 0.28) | < .001 |  |  |
| Indirect effect | 0.04 (0.02 – 0.06) | < .001 |  | 0.17 | 0.03 (0.01 – 0.05) | .001 |  | 0.14 |
| **Bullying severity** |  |  | 452 |  |  |  | 452 |  |
| Total effect | 0.28 (0.19 – 0.37) | < .001 |  |  | 0.24 (0.15 – 0.33) | < .001 |  |  |
| Direct effect | 0.25 (0.16 – 0.33) | < .001 |  |  | 0.21 (0.12 – 0.30) | < .001 |  |  |
| Indirect effect | 0.03 (0.01– 0.05) | .001 |  | 0.11 | 0.03 (0.01 – 0.05) | .002 |  | 0.13 |

*Exploratory analyses of the indirect effect of childhood adversity on dimensions of prodromal symptoms (hallucinations vs. delusions) via pathways through threat anticipation*

*Note*. Threat anticipation assessed with the availability test. Childhood trauma assessed with the JVQ. Subscales of prodromal symptoms assessed with the PQ-16. aβ = results adjusted for age, gender, self-reported ethnicity, and cognitive deviance. CI = 95% confidence interval. *N_min_* = due to varying numbers of missing values, different paths of the mediation analyses comprised varying sample sizes. Therefore, the minimum sample size is displayed here. *P_M_* =

# **Overview of missing values**

**Table S5**

|  |  | *Missing values per scale* |
| --- | --- | --- |
| **Variables reported by adolescents** | |  |
| Age | | 0 |
| Gender | | 4 |
| Ethnicity | | 1,231 |
| Self-reported ethnicity | | 0 |
| Childhood adversity  Conventional crime ^a^  Indirect victimization  Child maltreatment  Peer or sibling victimization  Sexual victimization  Bullying prevalence  Cyber bullying prevalence  Physical bullying prevalence | | 1,077  425  369  370  376  441  449  455 |
| Threat anticipation | | 0 |
| Lifetime prevalence of prodromal symptoms ^a^ | | 1,024 |
| General psychopathology | | 326 |
| **Variables reported by parents/caregivers** | |  |
| Cohabitation | | 1,237 |
| Income | | 1,267 |
| Ethnicity  Educator 1  Educator 2 | | 1,239  1,269 |
| Education  Educator 1  Educator 2 | | 1,236  1,252 |
| Employment  Educator 1  Educator 2 | | 1,237  1,253 |

*Distribution of missing values across scales*

^a^ scale was omitted for 12-year-olds, resulting in missing values.

# **Sensitivity analyses**

## **Sensitivity analyses with restrictions on missing values**

In line with the manuals (Derogatis, 1993; Ising et al., 2012; Loewy, Bearden, Johnson, Raine, & Cannon, 2005), we allowed for one missing value per scale for the BSI and one missing on the PQ-16. The PQ-16 perceived distress variable had to be omitted as there were too many missing values as perceived distress was assessed only if the participant indicates that he or she has already experienced the symptom. No missing values allowed for threat anticipation.

### Hypothesis 1: The association of threat anticipation and psychopathology – with restrictions on missing values

**Table S6**

|  | General psychopathology | | | Prodromal symptoms Anomalous experiences | | | |
| --- | --- | --- | --- | --- | --- | --- | --- |
|  |  |  |  |  |  |  |  |
|  | aβ (CI) | *p* | *N* | | aβ (CI) | *p* | *N* |
| Threat anticipation | 0.49 (0.43 – 0.56) | < .001 | 1,112 | | 0.53 (0.36 – 0.69) | < .001 | 399 |

*Sensitivity analysis: The association of threat anticipation and psychopathology – with restrictions on missing values*

*Note*. Threat anticipation assessed with the availability test General psychopathology assessed with the BSI-53. Prodromal symptoms assessed with the PQ-16.. aβ = results adjusted for age, gender, self-reported ethnicity, and cognitive deviance. CI = 95% confidence interval.

### Hypothesis 2: The association of childhood adversity and psychopathology – with restrictions on missing values

**Table S7**

*Sensitivity analysis: The association of childhood adversity (i.e., childhood trauma, bullying prevalence, and bullying severity) and psychopathology – with restrictions on missing values*

|  | General psychopathology | | | Prodromal symptoms Anomalous experiences | | |
| --- | --- | --- | --- | --- | --- | --- |
|  |  |  |  |  |  |  |
|  | aβ (CI) | *p* | *N* | aβ (CI) | *p* | *N* |
| Childhood trauma | 0.52 (0.46 – 0.58) | < .001 | 834 | 0.47 (0.38 – 0.57) | < .001 | 322 |
| Bullying prevalence | 0.36 (0.29 – 0.43) | < .001 | 971 | 0.26 (0.16 – 0.36) | < .001 | 356 |
| Bullying severity | 0.43 (0.36 – 0.50) | < .001 | 981 | 0.29 (0.19 – 0.39) | < .001 | 358 |

*Note*. Childhood trauma assessed with the JVQ. General psychopathology assessed with the BSI-53. Prodromal symptoms assessed with the PQ-16. aβ = results adjusted for age, gender, self-reported ethnicity, and cognitive deviance. CI = 95% confidence interval.

### Hypothesis 3: The indirect effect of childhood adversity on psychopathology via pathways through threat anticipation – with restrictions on missing values

**Table S8**

*Sensitivity analysis: The indirect effect of childhood adversity on psychopathology via pathways through threat anticipation – with restrictions on
missing values*

|  | General psychopathology | | | | Prodromal symptoms  Anomalous experiences | | | |
| --- | --- | --- | --- | --- | --- | --- | --- | --- |
|  |  |  |  |  |  |  |  |  |
|  | aβ (CI) | *p* | *N_min_* | *P_M_* | aβ (CI) | *p* | *N_min_* | *P_M_* |
| **Childhood trauma** | | | 820 |  |  |  | 316 |  |
| Total effect | 0.52 (0.46 – 0.58) | < .001 |  |  | 0.48 (0.39 – 0.57) | < .001 |  |  |
| Direct effect | 0.40 (0.34 – 0.46) | < .001 |  |  | 0.38 (0.28 – 0.47) | < .001 |  |  |
| Indirect effect | 0.13 (0.10 – 0.16) | < .001 |  | 0.25 | 0.10 (0.05 – 0.15) | < .001 |  | 0.21 |
| **Bullying prevalence** | |  | 870 |  |  |  | 337 |  |
| Total effect | 0.37 (0.31 – 0.43) | < .001 |  |  | 0.28 (0.18 – 0.37) | < .001 |  |  |
| Direct effect | 0.28 (0.23 – 0.34) | < .001 |  |  | 0.17 (0.08 – 0.27) | < .001 |  |  |
| Indirect effect | 0.09 (0.06 – 0.11) | < .001 |  | 0.24 | 0.10 (0.06 – 0.14) | < .001 |  | 0.36 |
| **Bullying severity** | |  | 882 |  |  |  | 339 |  |
| Total effect | 0.44 (0.38 – 0.50) | < .001 |  |  | 0.32 (0.22 – 0.41) | < .001 |  |  |
| Direct effect | 0.35 (0.29 – 0.40) | < .001 |  |  | 0.21 (0.11 – 0.30) | < .001 |  |  |
| Indirect effect | 0.09 (0.06 – 0.12) | < .001 |  | 0.21 | 0.11 (0.07 – 0.15) | < .001 |  | 0.34 |

*Note*. Threat anticipation assessed with the availability test. Childhood trauma assessed with the JVQ. .General psychopathology assessed with the BSI-53. Prodromal symptoms assessed with the PQ-16. aβ = results adjusted for age, gender, self-reported ethnicity, and cognitive deviance. CI = 95% confidence interval. *N_min_* = due to varying numbers of missing values, different paths of the mediation analyses comprised varying sample sizes. Therefore, the minimum sample size is displayed here. *P_M_* = proportion mediated.

## **Sensitivity analyses after exclusion of outliers**

We identified 103 outliers based on an outlier analysis for skewed data (Hubert & Van der Veeken, 2008) and excluded them for this sensitivity analysis.

### Hypothesis 1: The association of threat anticipation and psychopathology – after exclusion of outliers

**Table S9**

|  | General psychopathology | | | Prodromal symptoms | | | | | |
| --- | --- | --- | --- | --- | --- | --- | --- | --- | --- |
|  |  |  |  | Anomalous experiences | | | Perceived distress | | |
|  | aβ (CI) | *p* | *N* | aβ (CI) | *p* | *N* | aβ (CI) | *p* | *N* |
| Threat anticipation | 0.35 (0.28 – 0.42) | < .001 | 1,300 | 0.31 (0.18 – 0.43) | < .001 | 567 | 0.39 (0.25 – 0.54) | < .001 | 567 |

*Sensitivity analysis: The association of threat anticipation and psychopathology – after exclusion of outliers*

*Note*. Threat anticipation assessed with the availability test. General psychopathology assessed with the BSI-53. Prodromal symptoms assessed with the PQ-16. aβ = results adjusted for age, gender, self-reported ethnicity, and cognitive deviance. CI = 95% confidence interval.

### Hypothesis 2: The association of childhood adversity and psychopathology – after exclusion of outliers

**Table S10**

*Sensitivity analysis: The association of childhood adversity (i.e., childhood trauma, bullying prevalence, and bullying severity) and psychopathology – after exclusion of outliers*

|  | General psychopathology | | | Prodromal symptoms | | | | | |
| --- | --- | --- | --- | --- | --- | --- | --- | --- | --- |
|  |  |  |  | Anomalous experiences | | | Perceived distress | | |
|  | aβ (CI) | *p* | *N* | aβ (CI) | *p* | *N* | aβ (CI) | *p* | *N* |
| Childhood trauma | 0.52 (0.45 – 0.59) | < .001 | 1,163 | 0.33 (0.23 – 0.44) | < .001 | 525 | 0.34 (0.24 – 0.44) | < .001 | 525 |
| Bullying prevalence | 0.31 (0.24 – 0.38) | < .001 | 971 | 0.27 (0.17 – 0.37) | < .001 | 416 | 0.27 (0.16 – 0.37) | < .001 | 416 |
| Bullying severity | 0.42 (0.34 – 0.50) | < .001 | 980 | 0.35 (0.24 – 0.47) | < .001 | 415 | 0.34 (0.22 – 0.46) | < .001 | 415 |

*Note*. Childhood trauma assessed with the JVQ. General psychopathology assessed with the BSI-53. Prodromal symptoms assessed with the PQ-16. aβ = results adjusted for age, gender, cognitive deviance, and self-reported ethnicity.. CI = 95% confidence interval.

### Hypothesis 3: Threat anticipation as a mediator of the association between childhood adversity and psychopathology – after exclusion of outliers

**Table S11**

### *Sensitivity analysis: The indirect effect of childhood adversity on psychopathology via pathways through threat anticipation – after exclusion of outliers*

|  | General psychopathology | | | | | | | | | | Prodromal symptoms | | | | | | | | | | |
| --- | --- | --- | --- | --- | --- | --- | --- | --- | --- | --- | --- | --- | --- | --- | --- | --- | --- | --- | --- | --- | --- |
|  |  | | |  | | | |  | | | Anomalous experiences | | | | | | Perceived distress | | | | |
|  | aβ (CI) | | | *p* | | | | *N_min_* | *P_M_* | | aβ (CI) | | *p* | *N_min_* | *P_M_* | | aβ (CI) | | *p* | *N_min_* | *P_M_* |
| **Childhood trauma** | | |  | | | |  | 1,163 |  | |  |  | | 525 |  |  | |  | | 525 |  |
| Total effect | 0.52  (0.47 – 0.57) | | | < .001 | | | |  |  | | 0.34  (0.27 – 0.42) | | < .001 |  |  | | 0.35  (0.28 – 0.43) | | < .001 |  |  |
| Direct effect | 0.40  (0.34 – 0.45) | | | < .001 | | | |  |  | | 0.27 (0.19 – 0.36) | | < .001 |  |  | | 0.25  (0.17 – 0.33) | | < .001 |  |  |
| Indirect effect | 0.12  (0.10 – 0.15) | | | < .001 | | | |  | 0.23 | | 0.07  (0.03 – 0.11) | | < .001 |  | 0.21 | | 0.10  (0.06 – 0.14) | | < .001 |  | 0.29 |
| **Bullying prevalence** | | | |  | |  | | 971 |  |  | |  | | 416 |  |  | |  | | 416 |  |
| Total effect | 0.31  (0.25 – 0.37) | | | < .001 | | | |  |  | | 0.27  (0.17 – 0.37) | | < .001 |  |  | | 0.27  (0.17 – 0.37) | | < .001 |  |  |
| Direct effect | 0.26 (0.21 – 0.32) | | | < .001 | | | |  |  | | 0.22  (0.13 – 0.32) | | < .001 |  |  | | 0.21  (0.12 – 0.31) | | < .001 |  |  |
| Indirect effect | 0.05  (0.03 – 0.07) | | | < .001 | | | |  | 0.16 | | 0.05  (0.02 – 0.07) | | < .001 |  | 0.19 | | 0.06  (0.03 – 0.08) | | < .001 |  | 0.22 |
| **Bullying severity** | |  | | |  | | | 980 |  |  | |  | | 415 |  |  | |  | | 415 |  |
| Total effect | 0.42  (0.35 – 0.49) | | | < .001 | | | |  |  | | 0.36  (0.25 – 0.46) | | < .001 |  |  | | 0.36  (0.25 – 0.47) | | < .001 |  |  |
| Direct effect | 0.36  (0.30 – 0.42) | | | < .001 | | | |  |  | | 0.30  (0.20 – 0.41) | | < .001 |  |  | | 0.29  (0.18 – 0.40) | | < .001 |  |  |
| Indirect effect | 0.06  (0.04 – 0.09) | | | < .001 | | | |  | 0.14 | | 0.05  (0.02 – 0.08) | | < .001 |  | 0.14 | | 0.06  (0.03 – 010) | | < .001 |  | 0.17 |

*Note*. Threat anticipation assessed with the availability test. Childhood trauma assessed with the JVQ. General psychopathology assessed with the BSI-53. Prodromal symptoms assessed with the PQ-16. aβ = results adjusted for age, gender, self-reported ethnicity, and cognitive deviance. CI = 95% confidence interval. *N_min_* = due to varying numbers of missing values, different paths of the mediation analyses comprised varying sample sizes. Therefore, the minimum sample size is displayed here. *P_M_* = proportion mediated.

## **Sensitivity analyses with restriction on missing values and after exclusion of outliers**

In line with the manuals (Derogatis, 1993; Ising, et al., 2012; Loewy, et al., 2005), we allowed for one missing value per scale for the BSI and one missing on the PQ-16. The PQ-16 perceived distress variable had to be omitted as there were too many missing values as perceived distress was assessed only if the participant indicates that he or she has already experienced the symptom. No missing values allowed for threat anticipation. We identified 103 outliers based on an outlier analysis for skewed data (Hubert & Van der Veeken, 2008) and excluded them for this sensitivity analysis.

### Hypothesis 1: The association of threat anticipation and psychopathology – with restrictions on missing values and after exclusion of outliers

**Table S12**

|  | General psychopathology | | | Prodromal symptoms Anomalous experiences | | | |
| --- | --- | --- | --- | --- | --- | --- | --- |
|  |  |  |  |  |  |  |  |
|  | aβ (CI) | *p* | *N* | | aβ (CI) | *p* | *N* |
| Threat anticipation | 0.51 (0.45 – 0.56) | < .001 | 1,045 | | 0.52 (0.36 – 0.69) | < .001 | 373 |

*Sensitivity analysis: The association of threat anticipation and psychopathology – with restrictions on missing values and after exclusion of outliers*

*Note*. Threat anticipation assessed with the availability test. General psychopathology assessed with the BSI-53. Prodromal symptoms assessed with the PQ-16. aβ = results adjusted for age, gender, self-reported ethnicity, and cognitive deviance. CI = 95% confidence interval.

### Hypothesis 2: The association of childhood adversity and psychopathology – with restrictions on missing values and after exclusion of outliers

**Table S13**

|  | General psychopathology | | | Prodromal symptoms Anomalous experiences | | |
| --- | --- | --- | --- | --- | --- | --- |
|  |  |  |  |  |  |  |
|  | aβ (CI) | *p* | *N* | aβ (CI) | *p* | *N* |
| Childhood trauma | 0.52 (0.45 – 0.58) | < .001 | 790 | 0.47 (0.38 – 0.57) | < .001 | 302 |
| Bullying prevalence | 0.33 (0.25 – 0.40) | < .001 | 904 | 0.29 (0.18 – 0.40) | < .001 | 330 |
| Bullying severity | 0.44 (0.36 – 0.52) | < .001 | 909 | 0.41 (0.29 – 0.53) | < .001 | 329 |

*Sensitivity analysis: The association of childhood adversity (i.e., childhood trauma, bullying prevalence, and bullying severity) and psychopathology – with restrictions on missing values and after exclusion of outliers*

*Note*. Childhood trauma assessed with the JVQ. General psychopathology assessed with the BSI-53. Prodromal symptoms assessed with the PQ-16. aβ = results adjusted for age, gender, self-reported ethnicity, and cognitive deviance. CI = 95% confidence interval.

- - 1. Hypothesis 3: The indirect effect of childhood adversity on psychopathology via pathways through threat anticipation – with restrictions on missing values and after exclusion of outliers

**Table S14**

|  | General psychopathology | | | | Prodromal symptoms  Anomalous experiences | | | |
| --- | --- | --- | --- | --- | --- | --- | --- | --- |
|  |  |  |  | |  |  |  |  |
|  | aβ (CI) | *p* | *N_min_* | *P_M_* | aβ (CI) | *p* | *N_min_* | *P_M_* |
| **Childhood trauma** | | | 777 |  |  |  | 297 |  |
| Total effect | 0.51  (0.46 – 0.57) | < .001 |  |  | 0.48  (0.39 – 0.57) | < .001 |  |  |
| Direct effect | 0.39  (0.33 – 0.45) | < .001 |  |  | 0.38  (0.28 – 0.47) | < .001 |  |  |
| Indirect effect | 0.13  (0.10 – 0.16) | < .001 |  | 0.25 | 0.10  (0.05 – 0.15) | < .001 |  | 0.21 |
| **Bullying prevalence** | |  | 810 |  |  |  | 314 |  |
| Total effect | 0.34  (0.27 – 0.40) | < .001 |  |  | 0.31  (0.20 – 0.41) | < .001 |  |  |
| Direct effect | 0.25 (0.19 – 0.31) | < .001 |  |  | 0.21  (0.11 – 0.31) | < .001 |  |  |
| Indirect effect | 0.09  (0.06 – 0.12) | < .001 |  | 0.26 | 0.10  (0.06 – 0.13) | < .001 |  | 0.32 |
| **Bullying severity** | |  | 818 |  |  |  | 313 |  |
| Total effect | 0.46  (0.39 – 0.53) | < .001 |  |  | 0.43  (0.31 – 0.54) | < .001 |  |  |
| Direct effect | 0.35  (0.28 – 0.42) | < .001 |  |  | 0.31  (0.19 – 43) | < .001 |  |  |
| Indirect effect | 0.11  (0.07 – 0.14) | < .001 |  | 0.24 | 0.11  (0.07 – 0.16) | < .001 |  | 0.26 |

*Sensitivity analysis: The indirect effect of childhood adversity on psychopathology via pathways through threat anticipation – with restrictions on missing values and after exclusion of outliers*

*Note*. Threat anticipation assessed with the availability test. Childhood trauma assessed with the JVQ General psychopathology assessed with the BSI-53. Prodromal symptoms assessed with the PQ-16. aβ = results adjusted for age, gender, self-reported ethnicity, and cognitive deviance. CI = 95% confidence interval. *N_min_* = Due to varying numbers of missing values, different paths of the mediation analyses comprised varying sample sizes. Therefore, the minimum sample size is displayed here. *P_M_* = proportion mediated.

## **Sensitivity analyses with robust standard errors**

To account for heteroscedasticity in the computation of standard errors, we conducted sensitivity analyses with robust standard errors using the Huber/ White/ sandwich estimator (Freedman, 2006; Huber, 1967; White, 1980).

### Hypothesis 1: The association of childhood adversity and psychopathology – with robust standard errors

**Table S15**

*Sensitivity analysis: The association of childhood adversity and psychopathology – with robust standard errors*

|  | General psychopathology | | | Prodromal symptoms | | | | | |
| --- | --- | --- | --- | --- | --- | --- | --- | --- | --- |
|  |  |  |  | Anomalous experiences | | | Perceived distress | | |
|  | aβ (CI) | *p* | *N* | aβ (CI) | *p* | *N* | aβ (CI) | *p* | *N* |
| Threat anticipation | 0.36 (0.30 – 0.41) | < .001 | 1,384 | 0.28 (0.14 – 0.42) | < .001 | 607 | 0.35 (0.20– 0.51) | < .001 | 607 |

*Note*. Threat anticipation assessed with the availability test. General psychopathology assessed with the BSI-53. Prodromal symptoms assessed with the PQ-16. aβ = results adjusted for age, gender, self-reported ethnicity, and cognitive deviance. CI = 95% confidence interval.

### Hypothesis 2: The association of childhood adversity and psychopathology – with robust standard errors

**Table S16**

*Sensitivity analysis: The association of childhood adversity (i.e. childhood trauma, bullying prevalence and bullying severity) and psychopathology – with robust standard errors*

|  | General psychopathology | | | Prodromal symptoms | | | | | |
| --- | --- | --- | --- | --- | --- | --- | --- | --- | --- |
|  |  |  |  | Anomalous experiences | | | Perceived distress | | |
|  | aβ (CI) | *p* | *N* | aβ (CI) | *p* | *N* | aβ (CI) | *p* | *N* |
| Childhood trauma | 0.54 (0.47 – 0.61) | < .001 | 1,239 | 0.32 (0.22 – 0.43) | < .001 | 563 | 0.34 (0.24 – 0.44) | < .001 | 563 |
| Bullying prevalence | 0.35 (0.28 – 0.42)^a^ | < .001 | 1,045 | 0.23 (0.17 – 0.29) | < .001 | 449 | 0.24 (0.18 – 0.30) | < .001 | 449 |
| Bullying severity | 0.42 (0.35 – 0.49) | < .001 | 1,059 | 0.26 (0.17 – 0.35)^a^ | < .001 | 452 | 0.28 (0.20 – 0.36) | < .001 | 452 |

*Note*. Childhood trauma assessed with the JVQ. General psychopathology assessed with the BSI-53. Prodromal symptoms assessed with the PQ-16. aβ = results adjusted for age, gender, self-reported ethnicity, and cognitive deviance. CI = 95% confidence interval. ^a^ = the random intercept and random slope model cloud not be estimated, so the values are estimated with a random intercept model for this test.

### Hypothesis 3: The indirect effect of childhood adversity on psychopathology via pathways through threat anticipation

**Table S17**

*Sensitivity analysis: The indirect effects of childhood adversity (i.e., childhood trauma, bullying prevalence, and bullying severity) on psychopathology (i.e., general psychopathology, prodromal symptoms) via pathways through threat anticipation* *– with robust standard errors*

|  | General psychopathology | | | | | | | Prodromal symptoms | | | | | | | | | | | |
| --- | --- | --- | --- | --- | --- | --- | --- | --- | --- | --- | --- | --- | --- | --- | --- | --- | --- | --- | --- |
|  |  | | |  |  | | | Anomalous experiences | | | | | | Perceived distress | | | | | |
|  | aβ (CI) | | | *p* | *N_min_* | *P_M_* | | aβ (CI) | | *p* | *N_min_* | *P_M_* | | aβ (CI) | | *p* | *N_min_* | | *P_M_* |
| **Childhood trauma** | |  |  | | 1,239 |  |  | |  | | 563 |  |  | |  | | 563 |  | |
| Total effect | 0.55  (0.48 – 0.61) | | | < .001 |  |  | | 0.31  (0.21 – 0.42) | | < .001 |  |  | | 0.33  (0.23 – 0.44) | | < .001 |  | |  |
| Direct effect | 0.42  (0.35 – 0.49) | | | < .001 |  |  | | 0.26  (0.17 – 0.36) | | < .001 |  |  | | 0.25  (0.16 – 0.34) | | < .001 |  | |  |
| Indirect effect | 0.13  (0.10 – 0.15) | | | < .001 |  | 0.24 | | 0.05  (-0.00 – 0.10) | | .071 |  | 0.16 | | 0.08  (0.02 – 0.14) | | .008 |  | | 0.24 |
| **Bullying prevalence** | |  |  | | 1,045 |  |  | |  | | 449 |  |  | |  | | 449 |  | |
| Total effect | 0.35  (0.28 – 0.42) | | | < .001 |  |  | | 0.24  (0.17 – 0.30) | | < .001 |  |  | | 0.25  (0.19 – 0.31) | | < .001 |  | |  |
| Direct effect | 0.30  (0.23 – 0.37) | | | < .001 |  |  | | 0.20  (0.14 – 0.26) | | < .001 |  |  | | 0.20  (0.13 – 0.27) | | < .001 |  | |  |
| Indirect effect | 0.05  (0.02 – 0.07) | | | < .001 |  | 0.14 | | 0.04  (0.02 – 0.06) | | .011 |  | 0.17 | | 0.05  (0.02 – 0.07) | | < .001 |  | | 0.20 |
| **Bullying severity** | |  |  | | 1,059 |  |  | |  | | 452 |  |  | |  | | 452 |  | |
| Total effect | 0.41  (0.34 – 0.48) | | | < .001 |  |  | | 0.27  (0.18 – 0.36) | | < .001 |  |  | | 0.29  (0.22 – 0.37) | | < .001 |  | |  |
| Direct effect | 0.36  (0.29 – 0.44) | | | < .001 |  |  | | 0.24  (0.15 – 0.33) | | < .001 |  |  | | 0.25  (0.17 – 0.33) | | < .001 |  | |  |
| Indirect effect | 0.05  (0.03 – 0.07) | | | < .001 |  | 0.12 | | 0.04  (-0.00 – 0.8) | | .081 |  | 0.15 | | 0.05  (0.02 – 0.07) | | .001 |  | | 0.17 |

*Note*. Threat anticipation assessed with the availability test. Childhood trauma assessed with the JVQ. General psychopathology assessed with the BSI-53. Prodromal symptoms assessed with the PQ-16. aβ = results adjusted for age, gender, self-reported ethnicity, and cognitive deviance. CI = 95% confidence interval. *N_min_* = due to varying numbers of missing values, different paths of the mediation analyses comprised varying sample sizes. Therefore, the minimum sample size is displayed here. *P_M_* = proportion mediated.

# **Unadjusted analyses**

## **Hypothesis 1: The association of threat anticipation and psychopathology – unadjusted**

**Table S18**

|  | General psychopathology | | | Prodromal symptoms | | | | | |
| --- | --- | --- | --- | --- | --- | --- | --- | --- | --- |
|  |  |  |  | Anomalous experiences | | | Perceived distress | | |
|  | β (CI) | *p* | *N* | β (CI) | *p* | *N* | β (CI) | *p* | *N* |
| Threat anticipation | 0.39 (0.33 – 0.45) | < .001 | 1,476 | 0.32 (0.19 – 0.44) | < .001 | 658 | 0.39 (0.25 – 0.53) | < .001 | 658 |

*The association of threat anticipation and psychopathology* – *unadjusted*

*Note*. Threat anticipation assessed with the availability test. General psychopathology assessed with the BSI-53. Prodromal symptoms assessed with the PQ-16. CI = 95% confidence interval.

## **Hypothesis 2: The association of childhood adversity and psychopathology – unadjusted**

**Table S19**

|  | General psychopathology | | | Prodromal symptoms | | | | | | |
| --- | --- | --- | --- | --- | --- | --- | --- | --- | --- | --- |
|  |  |  |  | Anomalous experiences | | | Perceived distress | | | |
|  | β (CI) | *p* | *N* | β (CI) | *p* | *N* | β (CI) | *P* | *N* | |
| Childhood trauma | 0.54 (0.46 – 0.61) | < .001 | 1,319 | 0.35 (0.25 – 0.46) | < .001 | 607 | 0.36 (0.26 – 0.47) | < .001 | | 607 |
| Bullying prevalence | 0.37 (0.31 – 0.44) | < .001 | 1,112 | 0.26 (0.18 – 0.35) | < .001 | 484 | 0.27 (0.18 – 0.36) | < .001 | | 484 |
| Bullying severity | 0.43 (0.36 – 0.49) | < .001 | 1,128 | 0.29 (0.20 – 0.39) | < .001 | 488 | 0.31 (0.21 – 0.41) | < .001 | | 488 |

*The association of childhood adversity (i.e., childhood trauma, bullying prevalence, and bullying severity) and psychopathology*– *unadjusted*

*Note*. Childhood trauma assessed with the JVQ. General psychopathology assessed with the BSI-53. Prodromal symptoms assessed with the PQ-16. CI = 95% confidence interval.

- 1. **Hypothesis 3: The indirect effect of childhood adversity on psychopathology via pathways through threat anticipation**

**Table S20**

*The indirect effect of childhood adversity (i.e., childhood trauma, bullying prevalence, and bullying severity) on psychopathology (i.e., general psychopathology, prodromal symptoms) via pathways through threat anticipation*– *unadjusted*

|  | General psychopathology | | | | | | | Prodromal symptoms | | | | | | | | | | |
| --- | --- | --- | --- | --- | --- | --- | --- | --- | --- | --- | --- | --- | --- | --- | --- | --- | --- | --- |
|  |  | | | |  |  | | Anomalous experiences | | | | | | Perceived distress | | | | |
|  | β (CI) | | | | *p* | *N_min_* | *P_M_* | β (CI) | | *p* | *N_min_* | *P_M_* | | β (CI) | | *p* | *N_min_* | *P_M_* |
| **Childhood trauma** | |  |  |  | | 1,319 |  | |  | | 607 |  |  | |  | | 607 |  |
| Total effect | 0.53  (0.49 – 0.58) | | | | < .001 |  |  | 0.34  (0.27 – 0.40) | | < .001 |  |  | | 0.35 (0.28 – 0.41) | | < .001 |  |  |
| Direct effect | 0.41  (0.36 – 0.46) | | | | < .001 |  |  | 0.29 (0.22 – 0.36) | | < .001 |  |  | | 0.27  (0.20 – 0.34) | | < .001 |  |  |
| Indirect effect | 0.12  (0.10 – 0.15) | | | | < .001 |  | 0.23 | 0.05  (0.01 – 0.08) | | .007 |  | 0.15 | | 0.07  (0.04 – 0.11) | | < .001 |  | 0.20 |
| **Bullying prevalence** | |  |  |  | | 1,112 |  | |  | | 484 |  |  | |  | | 482 |  |
| Total effect | 0.41  (0.34 – 0.48) | | | | < .001 |  |  | 0.27  (0.19 – 0.35) | | < .001 |  |  | | 0.28  (0.20 – 0.36) | | < .001 |  |  |
| Direct effect | 0.31  (0.26 – 0.36) | | | | < .001 |  |  | 0.23  (0.15 – 0.31) | | < .001 |  |  | | 0.23  (0.15 – 0.31) | | < .001 |  |  |
| Indirect effect | 0.06  (0.04 – 0.08) | | | | < .001 |  | 0.15 | 0.05  (0.03 – 0.07) | | < .001 |  | 0.19 | | 0.05  (0.03 – 0.08) | | < .001 |  | 0.18 |
| **Bullying severity** | |  |  |  | | 1,128 |  | |  | | 488 |  |  | |  | | 488 |  |
| Total effect | 0.41  (0.36 – 0.46) | | | | < .001 |  |  | 0.30  (0.22 – 0.38) | | < .001 |  |  | | 0.32  (0.24 – 0.40) | | < .001 |  |  |
| Direct effect | 0.36  (0.31 – 0.41) | | | | < .001 |  |  | 0.26  (0.18 – 0.34) | | < .001 |  |  | | 0.27  (0.19 – 0.35) | | < .001 |  |  |
| Indirect effect | 0.05  (0.03 – 0.07) | | | | < .001 |  | 0.12 | 0.04  (0.02 – 0.06) | | < .001 |  | 0.13 | | 0.05  (0.03 – 0.07) | | < .001 |  | 0.16 |

*Note*. Threat anticipation assessed with the availability test. Childhood trauma assessed with the JVQ. General psychopathology assessed with the BSI-53. Prodromal symptoms assessed with the PQ-16. CI = 95% confidence interval. *N_min_* = due to varying numbers of missing values, different paths of the mediation analyses comprised varying sample sizes. Therefore, the minimum sample size is displayed here. *P_M_* = proportion mediated.

# **References**

Derogatis, L. R. (1993). *BSI brief symptom inventory. Administration, scoring, and procedures manual* (Vol. 4th ed.). Minneapolis, MN: National Computer Systems.

Freedman, D. A. (2006). On the so-called “Huber sandwich estimator” and “robust standard errors”. *The American Statistician, 60*, 299-302. doi: 10.1198/000313006X152207

Huber, P. J. (1967). *The behavior of maximum likelihood estimates under nonstandard conditions.* Paper presented at the Proceedings of the Fifth Berkeley Symposium on Mathematical Statistics and Probability: Weather modification.

Hubert, M., & Van der Veeken, S. (2008). Outlier detection for skewed data. *Journal of Chemometrics: A Journal of the Chemometrics Society, 22*, 235-246. doi: 10.1002/cem.1123

Ising, H. K., Veling, W., Loewy, R. L., Rietveld, M. W., Rietdijk, J., Dragt, S., . . . van der Gaag, M. (2012). The Validity of the 16-Item Version of the Prodromal Questionnaire (PQ-16) to Screen for Ultra High Risk of Developing Psychosis in the General Help-Seeking Population. *Schizophrenia Bulletin, 38*, 1288-1296. doi: 10.1093/schbul/sbs068

Loewy, R. L., Bearden, C. E., Johnson, J. K., Raine, A., & Cannon, T. D. (2005). The prodromal questionnaire (PQ): Preliminary validation of a self-report screening measure for prodromal and psychotic syndromes. *Schizophrenia Research, 79*, 117-125. doi: 10.1016/j.schres.2005.03.007

Simes, R. J. (1986). An improved Bonferroni procedure for multiple tests of significance. *Biometrika, 73*, 751-754. doi: 10.1093/biomet/73.3.751

White, H. (1980). A heteroskedasticity-consistent covariance matrix estimator and a direct test for heteroskedasticity. *Econometrica*, 817-838. doi: 10.2307/1912934
